# Supplementary material for: Development of the life change adaptation scale for family caregivers of individuals with acquired brain injury
Source: PLoS One. 2020 Oct 29;15(10):e0241386. doi: 10.1371/journal.pone.0241386 (PMC7595313; doi:10.1371/journal.pone.0241386)
Supplement: S1 Appendix — (PDF) [file pone.0241386.s001.pdf]

The life change adaptation scale for family caregivers of individuals with acquired brain injury (LCAS), English version

How your life or attitude has changed since acquired brain injury struck your family? Please check the rating closest to your opinion for each statement.

		The most deteriorated			Did not change			The most improved
Ex.	Your attitude of being considerate of the circumstances and feelings of others	-3	-2	-1	0	+ 1 ✓	+ 2	+ 3
1	Your attitude of being considerate of the circumstances and feelings of others	-3	-2	-1	0	+ 1	+ 2	+ 3
2	Your view toward social systems related to health and life for individuals with acquired brain injury	-3	-2	-1	0	+ 1	+ 2	+ 3
3	Your attitude of seeking help when needed	-3	-2	-1	0	+ 1	+ 2	+ 3
4	Your sense of responsibility as a member of family	-3	-2	-1	0	+ 1	+ 2	+ 3
5	Your attitude of not trying too hard alone on any issue	-3	-2	-1	0	+ 1	+ 2	+ 3
6	Your relaxed mind that allows enjoyment of leisure activities and hobbies	-3	-2	-1	0	+ 1	+ 2	+ 3
7	Your mindset of respecting your own health	-3	-2	-1	0	+ 1	+ 2	+ 3
8	Your outlook on your life going forward	-3	-2	-1	0	+ 1	+ 2	+ 3

Shindo Y, Tadaka E : Development of the life change adaptation scale for family caregivers of individuals with acquired brain injury, PLOS ONE (2020).
