# Supplementary material for: Development of the life change adaptation scale for family caregivers of individuals with acquired brain injury
Source: PLoS One. 2020 Oct 29;15(10):e0241386. doi: 10.1371/journal.pone.0241386 (PMC7595313; doi:10.1371/journal.pone.0241386)
Supplement: S2 Appendix — (PDF) [file pone.0241386.s002.pdf]

S2 Appendix. The LCAS Japanese Version.

The life change adaptation scale for family caregivers of individuals with acquired brain injury (LCAS), Japanese version

高次脳機能障害者の家族介護者における生活変化適応尺度（LCAS）日本語版

以下の各項目は、高次脳機能障害発生前後にご家族が経験する生活上の変化を表しています。

あなたのご家族に高次脳機能障害が生じたことで、あなたの生活ならびに姿勢等はどのように変わりましたか。
もっとも当てはまる数字１つに〇をつけてください。

		この上なく悪くなった			変わりない			この上なくよくなった
例	他者の立場や気持ちを考える姿勢	−3	−2	−1	0	＋1 (〇)	＋2	＋3
1	他者の立場や気持ちを考える姿勢	−3	−2	−1	0	＋1	＋2	＋3
2	ご本人の健康や生活に関わる社会制度への臨み方	−3	−2	−1	0	＋1	＋2	＋3
3	困った時は人の手を借りようとする姿勢	−3	−2	−1	0	＋1	＋2	＋3
4	家族の一員としての役割意識	−3	−2	−1	0	＋1	＋2	＋3
5	何事も一人で頑張りすぎない姿勢	−3	−2	−1	0	＋1	＋2	＋3
6	趣味などの余暇活動を楽しむゆとり	−3	−2	−1	0	＋1	＋2	＋3
7	自分自身の健康を大切にする心掛け	−3	−2	−1	0	＋1	＋2	＋3
8	この先の自分の生活に対する見通し	−3	−2	−1	0	＋1	＋2	＋3

Shindo Y, Tadaka E : Development of the life change adaptation scale for family caregivers of individuals with acquired brain injury, PLOS ONE (2020).
