# Supplementary material for: Development of the life change adaptation scale for family caregivers of individuals with acquired brain injury
Source: PLoS One. 2020 Oct 29;15(10):e0241386. doi: 10.1371/journal.pone.0241386 (PMC7595313; doi:10.1371/journal.pone.0241386)
Supplement: Attachment — Submitted filename: PONE Review June.docx [file pone.0241386.s003.docx]

PONE-D-20-15809

This study aimed to develop the Life Change Adaptation Scale (LCAS) for family caregivers of individuals with ABI and to examine its reliability and validity. This reviewer feels that the authors contribute something important to the wellbeing of family caregivers for persons with ABI. With the average length of care delivery being 12 years, a family member’s mental and physical health is paramount. This article a good fit for the PLOS One Journal with some revisions and copy editing.

Those suggestions are as follows:

- The 4 point scale anchors described in the text (1 = completely important, 2 = slightly important, 3 = slightly unimportant, 4 =not important at all) are not the same as appear on the English-language version of the instrument (a 7 point scale from “The most deterioration” to “did not change” to “The most improved”) which is a substantially different instrument than the one described.
- On that note, the factor names for factor 1 does not seem to reflect the items (factor 1=“Changes in the living resources for caregiving” and factor 2=“Changes in the health belief as a caregiver”). Factor one includes ‘Your attitude of being considerate to the circumstances and feelings of others”, “Your view toward social systems related to health and life for individuals with acquired brain injury”, “Your attitude of seeking help when needed”, and “Your sense of responsibility as a family member.”—all of the items seem to assess attitudinal changes and not changes in resources—perhaps changes in APPRAISAL of resources?
- Throughout, there are some minor semantic updates required—all of which are likely attributable to the translation from Japanese.
- The methods overview in the abstract on page 2, line 28-29 doubles uses the word confirmed/confirmatory which adds redundancy. Likewise, in the conclusions overview on page 3, lines 41-42, the first sentence uses “family caregivers of individuals with ABI” twice.
- On page 3, lines 43 and 44, the sentence that reads “This study contributes toward assessing the life change adaptation and identifying family caregivers of individuals with ABI who require intervention.” could be stronger (e.g., this study contributes to the assessment and identification…”)
- On page 5, lines 82-83, the authors note that caregivers of stroke victims are often older age rather than middle age, and that ABI caretakers are often middle aged or older. This is important because the author references the ‘life course and roles of the family caregiver’. It may be useful to define what ‘middle aged’ and ‘older aged’ means in this context.
- On page 8, lines 138-140, there is another repeated use of ‘family caregivers of individuals with ABI’.
- On page 9, line 149, the authors refer to “type of disorder” but they likely mean impairment? In table 2 on page 14, the factors listed range from attention, to topographical, to physical awareness.
- On page 20, lines 286-287, there is a repeated use of the word strong/strongly. On the same page, line 289, the sentence that reads “lacked of personal time for self-care” could better be worded as “lacked personal time for self-care”.
- Page 20, the final sentence ending on line 295, is powerful and a great way to end the paper. The section on limitations that follows should precede that section to maximize impact.
- One other limitation to note is the overrepresentation of female respondents (76%) and male ABI patients (81%)—this does represent the traditional dynamics but may contaminate assumptions about the meaning of the factors.
- In PLOS ONE, the P value should be expressed as a capital P—that change can be made throughout.
