# Supplementary material for: Development of the life change adaptation scale for family caregivers of individuals with acquired brain injury
Source: PLoS One. 2020 Oct 29;15(10):e0241386. doi: 10.1371/journal.pone.0241386 (PMC7595313; doi:10.1371/journal.pone.0241386)
Supplement: Attachment — Submitted filename: LCAS review.docx [file pone.0241386.s004.docx]

REVIEW: Major Revision

**Development of the Life Change Adaptation Scale for family caregivers of individuals with acquired brain injury**

**This manuscript will be of great interest to the field of ABI. I thank the researchers for undertaking this important work. I would be very pleased to read the revised draft as I think there is merit in this research but conceptual issues are present in the current version. Further revisions are needed with the key issues identified below:**

**Background**

Information about the incidence of strokes across all age groups is not included. These need to be more clearly defined or the statements substantiated with more evidence. This is an important issue as the patients with stroke and their families have been excluded.

Given the argument for caregivers being within a defined age – was a pediatric ABI population considered? If not, then the nature of caring for an adult that was previously independent needs to be included in the background.

What is the intention of developing this measure? What are the benefits for having such a measure for health services, health professionals and families? The rationale needs to be made clearer for what is missing and what the anticipated benefits.

**Results**

Why did some families receive the questionnaire that were not eligible?

Low response rate – please comment

Co-morbidities? Given the age range of the person with ABI (up to 76 years), were other aspects of health/disability and care required taken into consideration? Difficult to determine how much of the family adjustment process is due in part to the ageing process for the person with the ABI and other family members.

**Tables 3 and 4:**

These tables are not legible and therefore the reviewer is unable to make comment.

**Discussion**

The discussion needs to address the issues of ABI versus ageing. What was found between the ageing groups? Perhaps the analysis should be more about the comparison of groups by age or groups by time since injury

This measure has not addressed the changes related to the onset of an ABI – there are too many variables that have not been taken into account Or addressed in this section.
