# Supplementary material for: Development of the life change adaptation scale for family caregivers of individuals with acquired brain injury
Source: PLoS One. 2020 Oct 29;15(10):e0241386. doi: 10.1371/journal.pone.0241386 (PMC7595313; doi:10.1371/journal.pone.0241386)
Supplement: Attachment — Submitted filename: PONE Review 2.docx [file pone.0241386.s006.docx]

This reviewer sees that the authors have responded to each of the previous suggestions made and believe the changes made serve the paper well. Overall, the minor grammatical issues do seem to be remedied by the proofreader. A few additional copy edits include:

Line 65 should read “these individuals can achieve.” On the same line through 66 it is unclear what “this measure” is referring to.

On line 122 PsychInfo is improperly referred to as “Psycho-Info”.

Line 130 is missing a period after “22 items”.

Line 240 could benefit from copy editing (“The percentage of at least either one of response is higher than 85% of the sample.)

The clarification of how middle-age and older-age differ in developmental tasks in lines 81-84 does serve to improve understanding of the life course and roles of the family caregiver. Additionally, on line 77 older age is defined as above 65 years. Adult age is defined on line 78 as 16-44 years. Adding an age range for middle-age individuals is suggested here on line 79.

Before publication, it may be useful to describe the reason for including the K6 in more detail. Line 171-172 describes its use to validate the LCAS scale, however the K6 only assesses clinically significant serious mental illness. Therefore, an additional explanation of how this validates the LCAS, which includes positive aspects of mental health and the caregiving experience, seems necessary. Line 272 does state that these results are negatively correlated, but again could use clarification earlier. Additionally, the K6 in the English language version is scored from 1-5 where in line 173 the authors state, “each question is scored from 0 to 4”.

This reviewer believes that this paper presents the results of original research and that the results have not been published elsewhere.

This researcher believes that ethical and community standards are met in this paper. With very few final edits, this paper seemingly is suitable for publication in PLOS ONE.
