# Supplementary material for: Development of the life change adaptation scale for family caregivers of individuals with acquired brain injury
Source: PLoS One. 2020 Oct 29;15(10):e0241386. doi: 10.1371/journal.pone.0241386 (PMC7595313; doi:10.1371/journal.pone.0241386)
Supplement: Attachment — Submitted filename: Response to Reviewers.docx [file pone.0241386.s007.docx]

Dr. Manuel Fernández-Alcántara

Editor
*PLOS ONE*

October 8, 2020

Dear Dr. Fernández-Alcántara,

Thank you very much for your e-mail regarding our manuscript, “Development of the life change adaptation scale for family caregivers of individuals with acquired brain injury” (PONE -D-20-15809). We are grateful to know that it is potentially acceptable for publication in *PLOS ONE*. Please find attached a revised version of our manuscript.

Your comments and those of the reviewers were highly insightful and enabled us to greatly improve the quality of our manuscript. We include below our point-by-point responses to each of the comments of the reviewers as well as your own comments.

We look forward to hearing from you regarding our re-submission. We would be happy to respond to any further questions and comments that you may have.

Respectfully yours,

Yuka Shindo, MA, Etsuko Tadaka, PhD

Department of Community Health Nursing, Graduate School of Medicine, Yokohama City University

3-9, Fukuura, Kanazawa-ku, Yokohama, Kanagawa, 236-0004, Japan

Tel & Fax +81-45-787-2973

E-mail t206705c@yokohama-cu.ac.jp

**Response to Reviewers**

**To the comments of Reviewer #1**

1. *Line 65 should read “these individuals can achieve.” On the same line through 66 it is unclear what “this measure” is referring to.*

**Response:** “This measure” refers to measuring the life change adaptations. We have revised the text as follows (line 63-65 and line 66-67):

Line 63-65

“However, the family caregivers of individuals with ABI can experience not only negative adaptations to life changes but also positive adaptations following the onset of ABI, and such positive life change adaptations are just one of the benefits these individuals can achieve.”

Line 66-67

“Measuring the life change adaptations makes it possible to evaluate family caregivers of individuals with ABI and the health services offered by health professionals.”

2. *On line 122 PsychInfo is improperly referred to as “Psycho-Info”.*

**Response:** We have written it according to the American Psychological Association (*https://www.apa.org/pubs/databases/psycinfo/*) (line 122-123).

3. *Line 130 is missing a period after “22 items”.*

**Response:** We have added a period after “22 items” (line 130).

4. *Line 240 could benefit from copy editing (“The percentage of at least either one of response is higher than 85% of the sample.)*

**Response:** We have corrected the text as follows (line 248):

Line 248

“Item with a score (-3 to +3) of 85% or higher in the sample.”

5. *The clarification of how middle-age and older-age differ in developmental tasks in lines 81-84 does serve to improve understanding of the life course and roles of the family caregiver. Additionally, on line 77 older age is defined as above 65 years. Adult age is defined on line 78 as 16-44 years. Adding an age range for middle-age individuals is suggested here on line 79.*

**Response:** We considered ‘middle-aged’ to be 45-64 years old. We have now added the age range for middle-aged individuals as follows (line 78-81):

Line 78-81

“While the family caregivers of individuals with stroke are often in older age rather than middle-aged individuals (45-64years) [22], the family caregivers of individuals with ABI are often middle-aged or older.”

6. *Before publication, it may be useful to describe the reason for including the K6 in more detail. Line 171-172 describes its use to validate the LCAS scale, however the K6 only assesses clinically significant serious mental illness. Therefore, an additional explanation of how this validates the LCAS, which includes positive aspects of mental health and the caregiving experience, seems necessary. Line 272 does state that these results are negatively correlated, but again could use clarification earlier. Additionally, the K6 in the English language version is scored from 1-5 where in line 173 the authors state, “each question is scored from 0 to 4”. The 4 point scale anchors described in the text (1 = completely important, 2 = slightly important, 3 = slightly unimportant, 4 =not important at all) are not the same as appear on the English-language version of the instrument (a 7 point scale from “The most deterioration” to “did not change” to “The most improved”) which is a substantially different instrument than the one described.*

**Response:** The K6 assesses clinically significant serious mental illness using the optimal cut-off point, as the reviewer suggested. In addition, a previous study considered the K6 to assess the mental health using a continuous scale, with higher scores reflecting a poorer mental health (Oshio and Kan, 2016). Another previous study further cited a correlation between caregivers’ mental health and the perception of life changes (Negarandeh et al., 2015). Based on the findings from previous studies, we hypothesized that mental health deterioration in cases with higher K6 scores reflected negative life change adaptation with lower LCAS scores. We have now clarified how the K6 validates the LCAS (line 178-184) and corrected the explanation concerning validity in the text (line 280-282). In addition, we apologize for the confusion concerning the 4-point scale for the K6. We used the Japanese version of the K6 developed by Furukawa et al. [35], which is scored from 0-4. We have now added explanations about the 4-point scale for the K6 (line 172-174).

Line 172-174

“Each question rated the frequency of distress symptoms. The responses were scored as follows: 0 = none of the time, 1 = a little of the time, 2 = some of the time, 3 = a lot of the time, 4 = all of the time; thus, the total score ranges from 0 to 24.”

Line 178-184

“A previous study considered the K6 to be a continuous scale in which higher scores reflect lower levels of mental health [36]. In addition, another study explained that there was a correlation between an adverse impact on caregivers’ mental health and the negative perception regarding life changes [5]. Based on the findings from previous studies, we predicted that mental health deterioration in cases with higher K6 scores reflected negative life change adaptation with lower LCAS scores. Thus, the author used this scale as an indicator of concurrent validity.”

Line 280-282

“A negative life change adaptation as well as a low LCAS score showed a significant amount of variance between mental health deterioration as well as a high K6 score (r=-0.50; P<0.001).”

**To the comments of** **Reviewer #2**

1. *Please re-write the sentences on lines 146-152 so that the procedure is clearer to the reader.*

**Response:** We have carefully corrected (line 147-151).

Line 147-151

“Before sending the survey questionnaires, the author sent informed consent letters to the administrators of all associations of family caregivers of individuals with ABI. Thirty-nine associations (47.5%) consented to participate in the study. The survey questionnaires were distributed to each participant by the staff of each association.”

2. *Table 1 – Change ‘relationship to ABI’ to ‘relationship to individual with ABI’ (so that the terminology is consistent with Table 2.*

**Response:** We have changed “relationship to ABI” to “relationship to individual with ABI” (p.14-15, Table 1).

3. *Table 3 is still not legible. Please amend.*

**Response:** We apologize for this difficulty and have now enlarged the letters in Table 3 (p.17-18, Table 3)

4. *Table 5 – align the factors to the left of the column*

**Response:** We have now corrected this text (p.20-2l, table 5).

5. *The discussion needs to address issues related to the majority of family members are aged 55-74 years and length of time since injury – and how this impacted on the findings regarding adaptation. This does impact on life issues that are impacting on the family. For instance, families who have parents in this age range are more likely to have adult children who are no longer dependent. This is likely to therefore impact on the adaptation of the family to the circumstances they face in relation to ABI.*

**Response:** As suggested, we have now discussed how the family members age and length of the time since injury influenced the findings regarding adaptation (line 314-324).

Line 314-322

“The majority of family members being 55-74 years old and the length of time since the injury being over 10 years were considered to have influenced the findings regarding adaptation. In general, families who have parents in this age range are more likely to have adult children who are no longer dependent. The family caregivers of individuals with ABI must fill the role of caring for an adult who is now dependent after having fulfilled their role of caring for their children. The family caregivers of individuals with ABI therefore need to fill a specific role that most family members never experience. This is likely to influence the adaptation of the family to the circumstances they face in relation to ABI.”

6. *Limitations also need to be included*

**Response:** We moved the limitations in response to a previous comment from reviewer #1. Therefore, we have added a new subsection for the limitations to the discussion (line 352).
